# The Efficacy of Calcium Hypochlorite and Peroxyacetic Acid Treatments in Inactivating Enterohemorrhagic *Escherichia coli* on Alfalfa Seeds and Sprouts

**DOI:** 10.3390/microorganisms13020306

**Published:** 2025-01-30

**Authors:** Myung-Ji Kim, Wim Dejonghe, Murli Manohar, Jinru Chen

**Affiliations:** 1 Department of Food Science and Technology, The University of Georgia, Griffin, GA 30223-1797, USA; myungji.kim@uga.edu; 2 Ascribe Bioscience, Ithaca, NY 14850, USA; wim@ascribe-bio.com (W.D.);

**Keywords:** *E. coli* O157:H7, *E. coli* O104:H4, seed disinfection

## Abstract

For several decades, recurring outbreaks of human gastrointestinal infections associated with contaminated sprouts have posed an enduring challenge, highlighting the necessity of controlling the etiological agents on contaminated sprout seeds. This study investigated the efficacy of calcium hypochlorite and peroxyacetic acid treatments in inactivating the cells of four enterohemorrhagic *Escherichia coli* (EHEC) isolates—*viz*. *E. coli* O157:H7 K4492, F4546, and H1730, as well as *E. coli* O104:H4 BAA-2326—on alfalfa seeds and sprouts. The 2–3 log CFU/g of EHEC cells inoculated to sprout seeds became undetectable (≤1.40 log CFU/g) after treatment with the two sanitizers, even with the enrichment steps. Sprouts grown from calcium hypochlorite- and peroxyacetic acid-treated seeds had mean EHEC populations that were 4.54–4.60 log CFU/g and 1.25–1.52 log CFU/g lower, respectively, compared to those on sprouts grown from the untreated control seeds. Significantly (*p* ≤ 0.05) different from one another, the mean populations of the four EHEC isolates on harvested sprout samples were in the descending order of *E. coli* O157:H7 K4492, F4546, H1730, and *E. coli* O104:H4 BAA-2326. The results suggest that both sanitizing treatments effectively suppressed EHEC growth on alfalfa seeds and sprouts, but their effectiveness was bacterial-isolate-dependent.

## 1. Introduction

Sprout-related outbreaks of gastrointestinal infection present a substantial challenge for individuals striving to maintain a healthy diet. The outbreaks of *E. coli* O26 infection in 2012, O121 infection in 2014, O157 infections in 2016, and O103 infections in 2020 were all associated with sprout consumption [1], demonstrating the recurrent nature of these outbreaks in the United States. Patients from multiple states were impacted by these outbreaks, and some developed severe symptoms like fever, abdominal cramps, diarrhea, and hemolytic uremic syndrome. In comparison, the alfalfa sprout is most frequently linked to outbreaks of *Salmonella* and pathogen *E. coli* infections [2]. Sprout-borne bacteria usually come from contaminated seeds, and cells of these bacteria flourish during sprouting when humidity and temperature are suitable for their growth [3]. Due to public health concerns, vulnerable groups like those with compromised immunity, the elderly, and young children are advised to avoid the consumption of raw sprouts [4]. 

Previously, the National Advisory Committee on Microbiological Criteria for Foods (NACMCF) issued recommendations for seed treatment to mitigate sprout-associated outbreaks, which included disinfecting sprout seeds before sprouting with Ca(OCl)_2_ at a concentration of 20,000 ppm [3]. Chlorine treatments are widely used for disinfecting fresh produce [2]. The treatments could disrupt protein synthesis in bacterial cells, initiate DNA damage that compromises DNA transformation, inhibit oxygen uptake, and lead to macromolecule leakage [5].

Peroxyacetic acid (PAA)-based oxidant sanitizers have been approved for use in fresh produce wash water by the U.S. Environmental Protection Agency. However, the agency has not provided information about using PAA to treat alfalfa seeds for sprout production. PAA is easy to use, has compatibility with diverse organic matter and pH levels, and does not generate harmful by-products [6].

Among “the Sustainable Development Goals” established by the United Nations in 2015 are “Good Health and Well-Being” and “Responsible Consumption and Production”, which underscore the importance of mitigating foodborne illnesses associated with sprout consumption to protect vulnerable populations and utilizing sustainable sanitization practices with global priorities for eco-friendly and responsible food production [7]. To control sprout-associated outbreaks of enterohemorrhagic *Escherichia coli* (EHEC) infection, researchers have assessed the efficacy of different sanitizers in inactivating EHEC cells experimentally inoculated on alfalfa seeds. However, limited studies have focused on the possible variation among bacterial isolates in adhering to sprout seeds and their susceptibility to sanitizing treatments. Moreover, most available studies inoculated sprout seeds with extremely high levels (from 6 to 9 logs) of pathogens before sanitizing treatments [8,9,10]. This study aimed to evaluate the attachment ability of individual EHEC isolates to alfalfa seeds, to assess the efficacy of two commonly used chemical sanitizers, Ca(OCl)_2_ or PAA, against individual EHEC isolates at relatively lower inoculation levels (at 2–3 log CFU/g) on alfalfa seeds and to determine whether the treatments negatively affect the seed germination rate and sprout yield.

## 2. Materials and Methods

### 2.1. Bacterial Isolates and Inoculums

Four EHEC isolates (*E. coli* O104:H4 BAA-2326, *E. coli* O157:H7 F4546, *E. coli* O157:H7 H1730, and *E. coli* O157:H7 K4492), previously obtained from the laboratory culture collections of Drs. Larry Beuchat and Mark Harrison at the University of Georgia, were used in this study. The isolates were maintained at −80 °C from the time that they were made available to our laboratory. Repeated laboratory culturing was avoided to maintain the original physiological characteristics of the original isolates. In the current study, tryptic soy agar (TSA) and tryptic soy broth (TSB) supplemented with nalidixic acid (NA) at 50 µg/mL (designated as NATSA and NATSB, respectively) were used to cultivate the *E. coli* cultures (MP Biomedicals, Santa Ana, CA, USA). The initial bacterial cultures were obtained by streaking frozen stocks containing 15% (*v*/*v*) glycerol (Thermo Fisher Scientific, Suwanee, GA, USA) on NATSA and incubating at 37 °C for 24 h. Following incubation, one colony of each culture was transferred to a fresh NATSA plate and incubated at 37 °C for 9 h. The culture was then transferred to NATSB and incubated at 37 °C for 16 h. The EHEC cell suspension was diluted to 5 log CFU/ml using phosphate-buffered saline (PBS, pH 7.4) to inoculate alfalfa seeds. The microbiological media used in this study were purchased from Becton, Dickinson, and Company (Sparks, MD, USA) unless specified.

### 2.2. Seed Inoculation

Alfalfa (*Medicago sativa*) seeds were purchased from Twilley Seed Company (Otis S. Twilley Seed Co., Inc., Hodges, SC, USA). The alfalfa seeds were selected for this study because of their frequent involvement in sprout-related outbreaks of human gastrointestinal infections. To remove the background microflora, alfalfa seeds (2 g per sample) were placed in 5 mL of 2% sodium hypochlorite (Clorox Co., Oakland, CA, USA) for 15 min. Sanitized seeds were neutralized with 5 mL of Dey–Engley (D/E) neutralizing broth to remove the residual chlorine, followed by rinsing twice, each time with sterile deionized water. Seeds were then placed on sterile weighing paper in a laminar flow hood to dry for 1 h. Dried seeds were placed in 20 mL of an inoculum, with a bacterial concentration of 10^5^ CFU/mL, and agitated for 1 h at 100 rpm on an orbital platform shaker (model 3520, Lab-Line Instruments, Melrose Park, IL, USA). Inoculated seeds were drained, and EHEC cells attached to the alfalfa seeds were enumerated using the plate count assay.

### 2.3. Preparation of Chemical Sanitizers

The Ca(OCl)_2_ and PAA solutions were prepared and used by following the manufacturer’s instructions. Granules of Drytec^®^ (0.58 g; Innovative Water Care, Alpharetta, GA, USA), a chlorine-based sanitizer, were dissolved in 20 mL of sterilized deionized water to make a 20,000 ppm Ca(OCl)_2_ solution (pH 6.8). A solution with 80 ppm Tsunam^®^ 100 (Ecolab, St. Paul, MN, USA), a PAA-based sanitizer, was prepared by mixing 10.5 µL of the product with 20 mL of sterile deionized water (pH 2.9). The concentration of the sanitizers was confirmed using the Chlorine Test Strips (Cole-Parmer, Vernon Hills, IL, USA) and Peracetic Acid Test strips (Micro Essential Laboratory Inc., Brooklyn, NY, USA), respectively. All solutions were promptly utilized upon preparation.

### 2.4. Seed Treatment and Sprouting

Alfalfa seeds were treated with 20 mL of the Ca(OCl)_2_ or PAA solution described above for 15 min and 30 min, respectively. Control seeds were treated in sterilized deionized water for the same duration. The treated seeds were drained, neutralized with 10 mL of D/E broth for 10 min, and rinsed twice, each with sterile deionized water, before being planted on 1% (*w*/*v*) water agar in sterile squared Petri dishes (Electron Microscopy Sciences, Hatfield, PA, USA). The Petri dishes were kept in a plastic container with wet sterile paper towels lining the bottom. The plastic containers were placed in an environment at 25 °C for 7 d without light exposure.

### 2.5. Microbiological Analysis

Sprout samples were collected throughout the 7 d sprouting process to assess EHEC populations. Five pieces of sprouts were selected for weight measurement on days 3, 5, and 7, while ten pieces were collected on day 1. Each collected sprout sample was placed in a 30 mL whirl-pak bag (Nasco, Fort Atkinson, WI, USA) with 5 mL of 0.1 M PBS (pH 7.4). The samples were ground with a pestle for 1 min. The sample homogenates obtained were 10-fold diluted when necessary, and 100 µL of each original or appropriately diluted sample was plated on NATSA and sorbitol MacConkey agar (SMAC) or MacConkey agar (MAC) in duplicate to quantify the populations of *E. coli* O157:H7 and *E. coli* O104:H4. The population differences between the treated and water control samples were used to express the efficacy of the sanitizing treatments. Enrichment assays were conducted when samples tested negative for *E. coli* O157:H7 or O104:H4 in the plate count assays, following Chapter 4A of the Bacteriological Analytical Manual [11].

### 2.6. Germination Percentage and Sprout Yield 

The germination percentage and sprout yield were assessed using previously described methods with modifications [12]. One hundred and twenty seeds were used for each treatment with forty for each replicate. The seeds were placed in individual Petri dishes with sterilized paper towels moistened with sterile water. The seeds were germinated for one week at 25 °C, with regular sprays of sterile deionized water. The sprout yield was determined by measuring the initial weight (1 g per sample) and the weight after the 7-day sprouting period. The germination percentage was determined by dividing the number of germinated seeds by the total seeds used and multiplying by 100. Sprout yield was calculated by dividing the weight of the sprouts (g) by the weight of the treated seeds (g).

### 2.7. Statistical Analysis

The experiment was replicated twice, with duplicate samples analyzed for each treatment. The data were entered into the general linear model and analyzed using SAS software (SAS Institute, Cary, NC, USA). Fisher’s least significant difference test was applied to differentiate the means at a 95% confidence interval.

## 3. Results

### 3.1. EHEC Attachment to Alfalfa Seeds and Populations After Sanitizing Treatments

**Figure 1:**
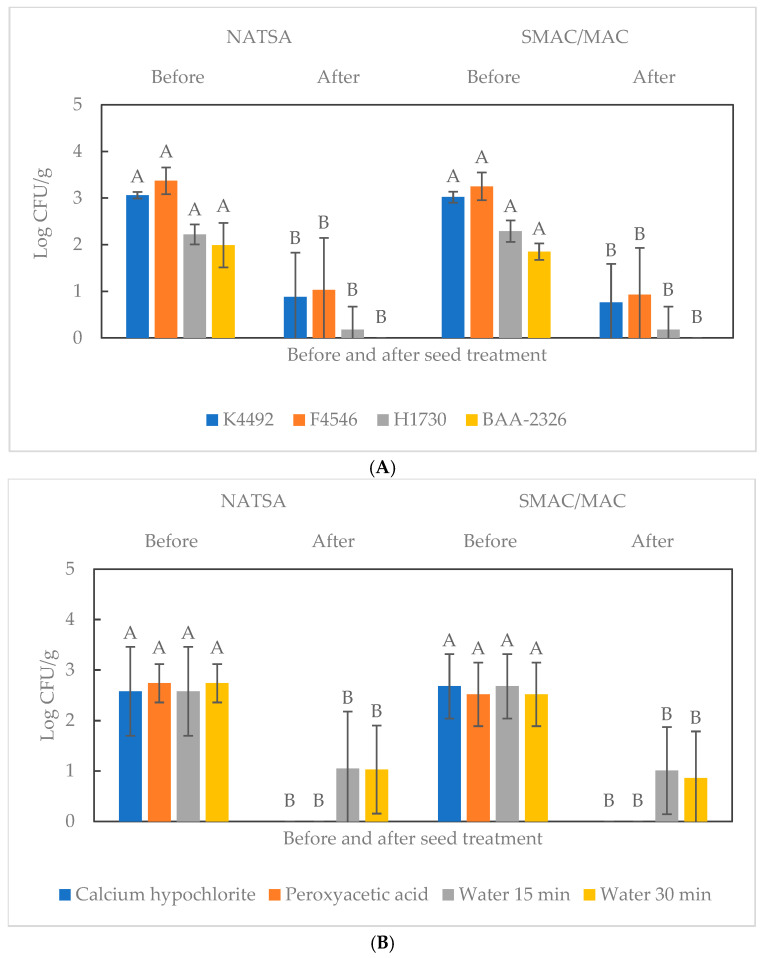
Cell populations of enterohemorrhagic *E. coli* (EHEC) on inoculated alfalfa seeds before and after sanitizing treatments. (**A**) Cell populations, separated by isolates, from tryptic soy agar amended with nalidixic acid (NATSA) or sorbitol MacConkey agar (SMAC)/MacConkey agar (MAC). (**B**) Cell populations, separated by treatment, from NATSA or SMAC/MAC. Bars with different letters are significantly different (*p* ≤ 0.05). Detection limit: ≤1.40 log CFU/g.

Figure 1 shows the levels of individual EHEC isolates attached to alfalfa seeds and the mean population of all four EHEC isolates on alfalfa seeds before and after the sanitizing treatments. The enumeration results obtained from both NATSA and SMAC/MAC show that the mean initial attachment levels of the four *E. coli* isolates followed a descending order of F4546, K4492, H1730, and BAA-2326, with the cell population ranging from 1.99 to 3.37 log CFU/g on NATSA and 1.85 to 3.25 log CFU/g on SMAC/MAC (Figure 1A). The same order in the EHEC cell population persisted after the sanitizing treatments, ranging from 0.93 or 1.03 log CFU/g to non-detectable levels (≤1.40 log CFU/g). Isolate BAA-2326 had the lowest levels of attachment to alfalfa seeds compared to the other three isolates used in the study, which reached non-detectable levels after the sanitizing and water control treatments. The plate count assay results indicated an average cell population reduction of *ca.* 2 log CFU/g for each EHEC isolate tested, with isolates with a higher initial attachment level being more tolerant to the sanitizing treatments (Figure 1A).

The results in Figure 1B illustrate the average cell populations of all four EHEC isolates before and after each sanitizing treatment. Ca(OCl)_2_ and PAA effectively reduced the average EHEC cell counts on alfalfa seeds to non-detectable levels on both types of growth media. After the water treatments, regardless of the treatment time, an average of *ca.* 1 log CFU/g of EHEC cells were recovered from the surface of alfalfa seeds (Figure 1B).

### 3.2. Reductions in EHEC Populations After Different Treatments

**Figure 2:**
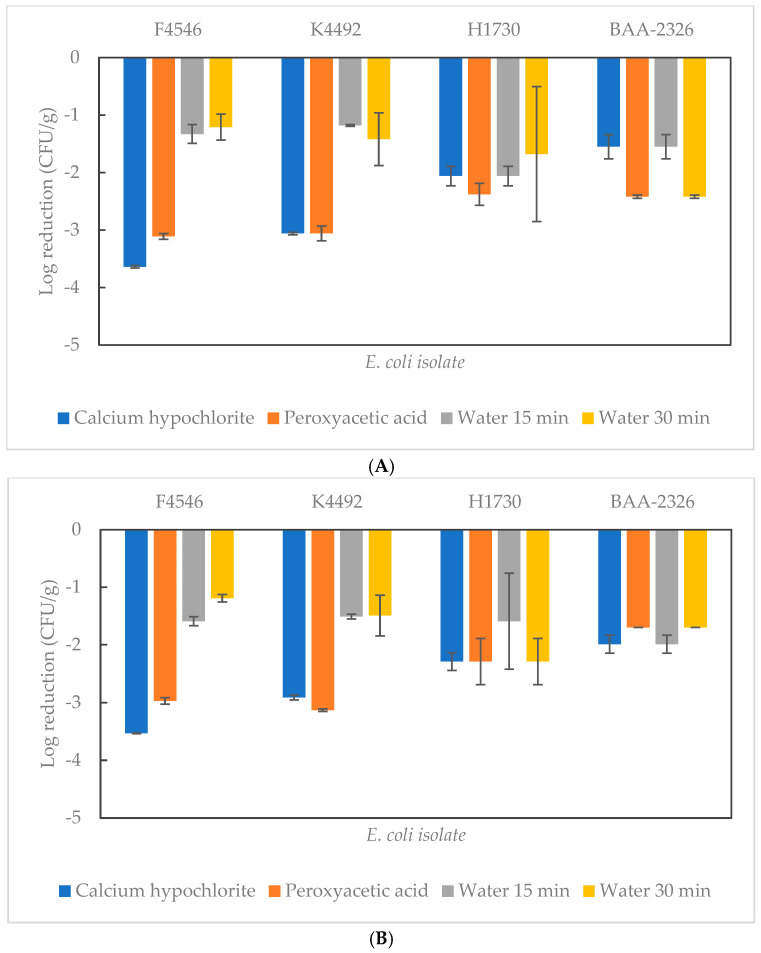
Inactivation of enterohemorrhagic *E. coli* (EHEC) on alfalfa seeds using different treatments. (**A**) Reduction in bacterial cell population obtained from tryptic soy agar supplemented with nalidixic acid (NATSA). (**B**) Reduction in EHEC cell populations from sorbitol MacConkey (SMAC) or MacConkey (MAC) agar.

Figure 2 presents the log reduction in each of the four EHEC isolates separated by each type of treatment and their corresponding controls. The Ca(OCl)_2_ and PAA treatments resulted in an average reduction in K4546 and F4492 populations by *ca.* 3 log CFU/g. In contrast, the levels of cell population reduction in water treatment control samples did not exceed 1.6 log CFU/g (Figure 2A). Specifically, treatment with Ca(OCl)_2_ achieved the highest reduction levels, with isolate F4546 showing a reduction level of 3.53 log CFU/g according to the results from SMAC (Figure 2B). The levels of cell population reductions of isolates H1730 and BAA-2326 did not differ significantly (*p* > 0.05) between the sanitizer-treated samples and the water treatment controls. The 20,000 ppm Ca(OCl)_2_ and 80 ppm PAA treatments reduced the populations of the two isolates by 2.29 log CFU/g based on the enumeration results on SMAC/MAC.

### 3.3. Results of Statistical Analysis of Data from Collected Sprout Samples 

Table 1 displays the average populations of individual EHEC isolates on alfalfa sprouts grown from seeds that received different types of treatments and of all four EHEC isolates at each sampling point or after each treatment. The mean populations of the four EHEC isolates differed significantly (*p* ≤ 0.05), with isolate K4492 having the highest average cell population, followed by F4546, H1730, and BAA-2326. The EHEC cell population increased with sprouting time until day 3 but did not exceed 2.50 log CFU/g on average. Treatments with Ca(OCl)_2_ and PAA reduced mean EHEC populations on sprouts by 4.54–4.60 log CFU/g and 1.25–1.52 log CFU/g, respectively, compared to their corresponding water treatment controls. While the efficacies of the treatments with Ca(OCl)_2_ and PAA were not significantly (*p* > 0.05) different from one another, the treatments with the two sanitizers were more effective than their water treatment controls. 

### 3.4. EHEC Populations of Individual EHEC Isolates Recovered on Alfalfa Sprouts Developed from Treated and Control Seeds 

The average populations of the four EHEC isolates on alfalfa sprouts grown from treated and control seeds from day 0 to day 7 are presented in Table 2. No EHEC cells were recovered from sprouts developed from alfalfa seeds inoculated with each of the four EHEC isolates and treated with Ca(OCl)_2_, as indicated by the non-detectable results from NATSA or SMAC/MAC and the negative results from the enrichment assay. 

The PAA seed treatment effectively controlled EHEC cell growth on alfalfa sprouts compared to its water treatment control (Table 2). Isolates F4546, H1730, and BAA-2326 were not detected on sprouts grown from PAA-treated seeds (Figure 1B); however, an average population of 1.28 log CFU/g for K4492 was detected on SMAC/MAC (Table 2). The cell populations of *E. coli* BAA-2326 were insignificantly (*p* > 0.05) different among the two sanitizer-treated groups and their control groups (Table 2). Excluding isolate BAA-2326, the Ca(OCl)_2_ and PAA treatments were significantly (*p* ≤ 0.05) more effective than the water controls in controlling the growth of F4546, K4492, and H1730 during sprouting (Table 2). 

### 3.5. Spout Yield and Germination Rate 

The sprout yield from treated and control seeds was not significantly different (*p* > 0.05). Similarly, the germination rates across all treatments were approximately 70%, also with no significant difference.

## 4. Discussion

### 4.1. EHEC Attachment to Sprout Seeds and Inactivation by Sanitizing Treatments 

In the current study, the attachment of EHEC cells to alfalfa seeds followed a descending order of F4546, K4492, H1730, and BAA-2326 (Figure 1A). Cui et al. also found that the cells of BAA-2326 had the lowest affinity to different types of vegetable seeds compared to the other three EHEC isolates used in the study [13]. Bacterial attachment to contact surfaces could be affected, in general, by both intrinsic and extrinsic factors, including the differences in bacterial cell surface structures, characteristics of cell–contact-surfaces, and the nature of their interaction. 

Cell surface appendages could influence bacterial attachment and colonization, and their effect depends on the host species or bacterial isolates involved [14]. Wang et al. reported that the number of adhesin genes carried by individual bacterial isolates affected the interaction between bacterial cells and their contact surfaces [15]. Macarisin et al. reported that curli, one of the bacterial adhesins, was vital in facilitating the adherence and endurance of *E. coli* O157:H7 on fresh produce [16]. They are involved in initial cell attachment to contact surfaces and aggregation among bacterial cells [17]. 

Adhesin production was not the sole determinant of cell attachment; other components, such as flagella and polysaccharides, were also involved [15,18]. *E. coli* O104:H4 BAA-2326 has the characteristics of enteroaggregative *E. coli* which are proliferating exopolysaccharide producers [19]. Some exopolysaccharides may have dual roles, inhibiting early cell attachment while enhancing cell aggregation during biofilm development [20]. The dual roles of these exopolysaccharides might be one of the contributing factors to the hindered attachment of BAA-2326 to alfalfa seeds. 

The initial step of bacterial attachment to surfaces can be influenced by factors such as electrostatic and hydrophobic interactions, hydrodynamic forces, etc. [21]. Previous studies showed that the surface of the alfalfa seed is smooth, lacks intricate shapes [22], and possesses a water-repelling waxy outer layer [23]. Dong et al. reported that the attachment of *E. coli* O157:H7 cells to their contact surfaces was influenced by epicuticular wax composition [24]. The level of negative charge on the cells of individual *E. coli* isolates combined with the hydrophobicity and surface topography of alfalfa seeds could determine the level of bacterial attachment. 

The treatments with Ca(OCl)_2_ or PAA reduced the mean population of all four EHEC isolates on alfalfa seeds to non-detectable levels, which the water treatment control failed to achieve (Figure 1B). Unlike sanitizing treatments which kill bacterial cells, a water wash can only dislodge attached bacterial cells from their contact surfaces [25]. It can also spread bacterial cells, cross-contaminating uncontaminated areas [26].

Taormina and Beuchat studied the efficacy of a 10 min treatment with 20,000 ppm Ca(OCl)_2_ or 80 ppm PAA in inactivating a five *E. coli* O157:H7 isolate mixture on alfalfa seeds [4]. With a similar inoculation level, the reduction in cell population was either comparable to or lower than that of the present study (Figure 1B), but the complete elimination of *E. coli* O157:H7 was not achieved. In contrast to the findings of the current study, Zhao et al. achieved a greater than 6 log CFU/g reduction in the population of a five isolate mixture of *E. coli* O157:H7 from seeds inoculated with 8 log CFU/g using 20,000 ppm Ca(OCl)_2_ for 0 (actual time of 20–30 sec) to 60 min [10]. Wang and Kniel achieved a 5.97 log CFU/g reduction in *E. coli* O104:H4 BAA-2326 cell population from an initial inoculation level of 9.63 log CFU/g after treatment with 20,000 ppm Ca(OCl)_2_ for 20 min [9]. However, the complete elimination of bacterial cells was not achieved.

Naturally contaminated sprout seeds usually harbor lower levels of bacterial cells than the artificially inoculated seeds used in some of the early studies [27]. The current study used alfalfa seeds with an initial inoculation level of 2–3 log CFU/g, the lowest cell population possible for this type of study. While the higher bacterial inoculation levels used in some of the previous studies enable insights into the maximal efficacies of chemical treatments, the use of lower levels allows the observation of more realistic levels of pathogen populations on sanitizer-treated seeds. The results of the current study revealed that alfalfa seeds treated with 20,000 ppm Ca(OCl)_2_ or 80 ppm PAA had non-detectable EHEC cell levels, even with the enrichment steps (Figure 1B).

In the current study, the average populations of H1730 and BAA-2326 on sanitizer-treated seeds were not significantly different from those on the control seeds (Figure 2); however, K4492 and F4546 had a significant (*p* ≤ 0.05) population difference of *ca*. 3 log CFU/g between sanitizer-treated and control samples (Figure 2). These observations could be attributed to the initial levels of the four EHEC isolates attached to alfalfa seeds (Figure 1A). Singh et al. reported that a lower inoculated population of *E. coli* O157:H7 allowed for the increased availability of sanitizer solutions to targeted bacterial cells than the higher inoculated populations [28]. Yet, when the inoculation level is lowered to *ca.* 2 log CFU/g, such as in the case of H1730 and BAA-2326, rinsing with water might be able to wash away the entire bacterial population on sprout seeds; thus, sanitizing efficacy like what was observed with K4492 and F4546 cannot be visualized. However, this observation does not endorse using water to remove bacterial contaminants from sprout seeds due to the lack of killing power of water rinsing and the pathogen-spreading possibility of seed rinsing water [25,26].

Besides the initial cell population on sprout seeds, the seed-to-sanitizer ratio should be considered to facilitate effective contact between bacterial cells and sanitizers. The current study used a ratio of 1:10 (wt/vol) for seeds to sanitizers, which eliminated EHEC cells on the surface of alfalfa seeds. Kumar et al. reported that a ratio of 1:4 or higher ratios of up to 1:10 of mung bean seeds to oxychloro-based sanitizer was effective in eliminating 3 to 4 log CFU/g of *E. coli* O157:H7 or *Salmonella* [29]. Taormina and Beuchat were, however, unable to eliminate 2 to 3.2 log CFU/g of *E. coli* O157:H7 on alfalfa seeds after a 15 min treatment with 20,000 ppm Ca(OCl)_2_ using a 1:4 seed-to-sanitizer ratio [4].

### 4.2. EHEC Population on Alfalfa Sprouts

Except for sprouts developed from seeds inoculated with K4492 and treated with PAA, those grown from all Ca(OCl)_2_-treated seeds and other PAA-treated seeds had non-detectable cell populations (Table 2). The reason why K4492 was recovered from collected sprout samples is not clear. However, this bacterial isolate has been reported to be the second-most robust in adherence among 58 evaluated *E. coli* O157:H7 isolates [30]. It might also have a stronger ability to recover from the sublethal injuries endured during seed sanitizer treatments and subsequently flourish during sprouting. 

One notable observation was that the cell populations of *E. coli* BAA-2326 were not significantly different among sprouts developed from seeds that received various types of treatments (*p* > 0.05), and those grown from seeds rinsed with water for 30 min also had non-detectable EHEC levels (Table 2). Wang and Kniel reported that the level of *E. coli* O104:H4 on sprouts grown from control seeds was 9.33 log CFU/g, only 0.65 log CFU/g higher than, although significantly different from, those grown from seeds treated with 20,000 ppm Ca(OCl)_2_ [9]. 

Based on the results of SMAC/MAC in the current study, treatments with Ca(OCl)_2_ and PAA reduced the mean EHEC populations on sprouts by 4.54 log CFU/g and 1.25 log CFU/g, respectively (Table 1). A previous study found that sprouts developed from seeds treated with Ca(OCl)_2_ and PAA had average *Salmonella* populations reduced by 4.85 and 4.67 log CFU/g, respectively, based on the enumeration results from bismuth sulfite agar, compared to the controls [31]. The EHEC populations peaked on sprouting day 3 at 2.37 to 2.47 log CFU/g in the current study (Table 1), while *Salmonella* populations peaked on day 5 at 4.78 to 4.80 log CFU/g. Using the same sprouting method but with different *Salmonella* and EHEC isolates, Hu et al. reported that populations of *E. coli* peaked on day 1, while *Salmonella* populations did not peak until day 3 [32]. It was suggested that *S. enterica* isolates could use sprout exudates more effectively [33], thus reaching higher cell populations on sprouts. Han et al. also reported that the *Salmonella* isolates exhibited a stronger affinity to sprouts than the EHEC isolates used in their studies [34]. Similarly, Barak et al. observed a relatively weak attachment of *E. coli* O157:H7 cells compared to the cells of other *E. coli* and *S. enterica* isolates used in the study, which made the attached cells of *E. coli* O157:H7 easily rinsed away with water [35].

### 4.3. Germination Rate and Sprout Yield 

In the current study, the germination rates of seeds treated with sanitizers and their controls were similar. Taormina and Beuchat also observed, when treating alfalfa seeds with 20,000 ppm Ca(OCl)_2_ for 10 min, a germination percentage of 70.7%, without a significant difference compared to the control group (77.0%) [4]. However, treatment with 80 ppm PAA for 10 min resulted in a relatively higher germination rate than treatment with 20,000 ppm Ca(OCl)_2_ at 85.0%, with their corresponding controls at 86.7%. In contrast to these results, Lang et al. observed a 90% germination rate after treating alfalfa seeds with 20,000 ppm Ca(OCl)_2_ for 15 min, with no significant difference from the water treatment control [8]. Weissinger and Beuchat also showed that 20,000 ppm Ca(OCl)_2_ treatment resulted in a 91.6% germination rate [36]. The reasons behind the varied germination rates in reported studies are not clear. However, variations in seed age and storage condition as well as the method of distribution may affect seed quality. The seeds used in the current study were purchased shortly before the experiments. However, the COVID-19 pandemic may have affected the security of freshly harvested seeds because of distribution constraints [37]. Nonetheless, the consistent germination rates between treated and control seeds imply a minimal effect of the sanitizer treatments. 

## 5. Conclusions

This study evaluated the efficacy of Ca(OCl)_2_ and PAA treatments in disinfecting alfalfa seeds experimentally contaminated with relatively realistic levels of individual isolates *E. coli* O157:H7 and *E. coli* O104:H4. Both treatments reduced the EHEC population on sprout seeds to non-detectable levels, demonstrating their effectiveness in sanitizing seeds contaminated with near-natural levels of pathogens. Sprouts grown from seeds treated with 20,000 ppm Ca(OCl)_2_ or 80 ppm PAA had mean EHEC populations that were 4.54–4.60 log CFU/g and 1.25–1.52 log CFU/g lower, respectively, than those developed from water-treated control seeds. Sprouts grown from Ca(OCl)_2_-treated seeds and most PAA-treated seeds had non-detectable EHEC cell populations, with the exception of the samples developed from seeds inoculated with K4492 and treated with PAA. The results underscore the challenges of controlling more robust and tolerant bacterial pathogens such as isolate K4492 on vegetable sprouts. Future studies are needed to optimize the treatment conditions or identify more effective mitigation approaches to control these pathogens.

## Figures and Tables

**Table 1:** Average populations of each enterohemorrhagic *E. coli* isolate, or all four isolates, on alfalfa sprouts grown from treated and control seeds, across sampling days, treatments, and replications.

	Population (Log CFU/g)	
	NATSA	SMAC/MAC
Isolate		
*E. coli* K4492 (n = 40)	2.58 ^A^	2.54 ^A^
*E. coli* F4546 (n = 40)	2.16 ^AB^	2.06 ^AB^
*E. coli* H1730 (n = 40)	1.64 ^B^	1.61 ^B^
*E. coli* BAA-2326 (n = 40)	0.20 ^C^	0.22 ^C^
Sampling time (day)		
3 (n = 32)	2.37 ^A^	2.47 ^A^
1 (n = 32)	1.99 ^A^	1.84 ^AB^
5 (n = 32)	1.68 ^A^	1.63 ^B^
7 (n = 32)	1.66 ^A^	1.64 ^B^
0 (n = 32)	0.52 ^B^	0.47 ^C^
Treatment		
Water 15 min (n = 40)	4.60 ^A^	4.54 ^A^
Water 30 min (n = 40)	1.75 ^B^	1.57 ^B^
Peroxyacetic acid (n = 40)	0.23 ^C^	0.32 ^C^
Calcium hypochlorite (n = 40)	ND ^C^	ND ^C^
Replication		
2 (n = 80)	1.71 ^A^	1.64 ^A^
1 (n = 80)	1.58 ^A^	1.58 ^A^

NATSA: tryptic soy agar supplemented with nalidixic acid; SMAC/MAC: sorbitol MacConkey agar/MacConkey agar. Values within each set of independent variables in the same column denoted with different letters are significantly different (*p* ≤ 0.05). ND: Non-detectable by standard plate count assays; detection limit: ≤1.40 log CFU/g.

**Table 2:** Average populations of four enterohemorrhagic *E. coli* isolates on alfalfa sprouts from treated and control seeds across all sampling days.

		Treatment			
	Isolate	Calcium Hypochlorite	Peroxyacetic Acid	Water 15 Min	Water 30 Min
NATSA (n = 80)	K4492	ND ^Ca, (−)^	0.92 ^Ca^	6.43 ^Aa^	2.97 ^Ba^
	F4546	ND ^Ca, (−)^	ND ^Ca, (−)^	6.83 ^Aa^	1.80 ^Ba^
	H1730	ND ^Ca, (−)^	ND ^Ca, (−)^	4.32 ^Ab^	2.22 ^Ba^
	BAA-2326	ND ^Aa, (−)^	ND ^Aa, (−)^	0.80 ^Ac^	ND ^Ab, (−)^
SMAC/MAC (n = 80)	K4492	ND ^Ca, (−)^	1.28 ^BCa^	6.27 ^Aab^	2.61 ^Ba^
	F4546	ND ^Ca, (−)^	ND ^Cb, (−)^	6.60 ^Aa^	1.64 ^Ba^
	H1730	ND ^Ca, (−)^	ND ^Cb, (−)^	4.41 ^Ab^	2.04 ^Ba^
	BAA-2326	ND ^Aa, (−)^	ND ^Ab, (−)^	0.89 ^Ac^	ND ^Ab, (−)^

NATSA: tryptic soy agar supplemented with nalidixic acid; SMAC/MAC: sorbitol MacConkey agar/MacConkey agar. Values in the same row followed by different uppercase letters are significantly different (*p* ≤ 0.05). Values in the same column followed by different lowercase letters are significantly different (*p* ≤ 0.05). ND: Non-detectable by standard plate count assays; detection limit: ≤1.40 log CFU/g. Enrichment results (+: positive; −: negative) are shown inside the parentheses.

## Data Availability

Data are available upon request.
